# Ozonation of Whole Blood Results in an Increased Release of Microparticles from Blood Cells

**DOI:** 10.3390/biom12020164

**Published:** 2022-01-21

**Authors:** Barbara Boczkowska-Radziwon, Piotr Józef Olbromski, Anna Rogowska, Magdalena Bujno, Marta Myśliwiec, Agnieszka Żebrowska, Dariusz Średziński, Barbara Polityńska, Marek Z. Wojtukiewicz, Piotr Radziwon

**Affiliations:** 1 Regional Center for Transfusion Medicine, 15-950 Bialystok, Poland; bradziwon@rckik.bialystok.pl (B.B.-R.); arogowska@rckik.bialystok.pl (A.R.); mbujno@rckik.bialystok.pl (M.B.); azebrowska@rckik.bialystok.pl (A.Ż.); dsredzinski@rckik.bialystok.pl (D.Ś.); 2 Department of Gynecology IV, Gynecology-Obstetrics University Hospital, 60-235 Poznan, Poland; olbromski.piotr@gmail.com; 3 Department of Oncology, Medical University of Bialystok, 15-027 Bialystok, Poland; marta.mysl@gmail.com (M.M.); mzwojtukiewicz@gmail.com (M.Z.W.); 4 Department of Psychology and Philosophy, Medical University of Bialystok, 15-950 Bialystok, Poland; bpolitynska@wp.pl; 5 Robinson College, University of Cambridge, Cambridge CB3 9AN, UK; 6 Department of Clinical Oncology, Comprehensive Cancer Center, 15-950 Bialystok, Poland; 7 Department of Hematology, Medical University of Bialystok, 15-276 Bialystok, Poland

**Keywords:** ozone, microparticles, autohemotherapy, blood coagulation

## Abstract

Autohemotherapy with ozonated blood is used in the treatment of a broad spectrum of clinical disorders. Ozone demonstrates strong oxidizing properties and causes damage to cell membranes. The impact of whole-blood ozonation on the release of microparticles from blood and endothelial cells and the concentration of selected markers in the hemostatic system (APTT, PT, D-dimer, fibrinogen) were investigated. Venous blood, obtained from 19 healthy men, was split into four equal parts and treated with air, 15 µg/mL ozone, or 30 µg/mL ozone, or left untreated. The number and types of microparticles released were determined using flow cytometry on the basis of surface antigen expression: erythrocyte-derived microparticles (CD235^+^), platelet-derived microparticles (CD42^+^), leukocyte-derived microparticles (CD45^+^), and endothelial-derived microparticles (CD144^+^). The study is the first to demonstrate that ozone induces a statistically significant increase in the number of microparticles derived from blood and endothelial cells. Although statistically significant, the changes in some coagulation factors were somewhat mild and did not exceed normal values.

## 1. Introduction

Ozone (O_3_) is an allotrope of oxygen composed of three oxygen atoms. It is a natural component of the atmosphere produced by lightning or ultraviolet irradiation. Ozone is quickly and spontaneously decomposed into O_2_ and a single oxygen atom (O). It is the strongest naturally occurring oxidant. Its effects include the generation of reactive oxygen species (e.g., superoxide or hydroxyl radicals, singlet oxygen), as well as free radicals, both of which induce oxidative stress [1].

Ozone gas is used in medicine via topical application, but it is also administered directly intravenously, intra-articularly, or via a method called autohemotherapy. In the procedure of autohemotherapy, a small amount of the patient’s venous blood (approximate ranges for safe blood collection are 1.2 mL/kg to 1.3 mL/kg, usually 50–100 mL) is collected into an ozone-resistant container [2]. The recommended anticoagulant is citrate dextrose solution A (ACD-A; USP: 2.13% free citrate ion) or sodium citrate (3.8% 10 mL per 100 mL of blood). Generally, heparin is not advisable, because it can induce thrombocytopenia. The oxygen/ozone mixture is then bubbled into the blood, gently mixed, and reinfused into the vein of the patient. Thus, ozone does not enter the blood circulation. Its effects are mediated by the formation of secondary messengers and the induction of a further adaptive response from the body in a hermetic dose–response relationship. Through this indirect mechanism of action, ozone stimulates adaptive mechanisms that can induce modulations in the organism by affecting the immune system, blood flow, and oxygenation, as well as oxidative stress [3,4,5].

The number of treatment sessions and the ozone dosage administered depend on the general condition of the patient, their age, and any underlying disease. From the clinical point of view, improvement in the patient’s condition occurs between the fifth and tenth sessions. The treatment is given daily if necessary. It may also be administered two to three times a week.

Autohemotherapy with ozonated blood has been used to treat a wide variety of diseases, including heart disease, ischemic diseases (peripheral arterial, ocular circulatory and cerebral circulatory disturbances), pain management, diabetic angiopathy, trophic skin lesions, arthritis, herpes zoster infection, cancer (complementary treatment), hepatitis, and bacterial infection (complementary therapy) [6,7,8,9]. 

Ozone therapy is considered to be relatively safe. Nevertheless, there are several reports presenting adverse reactions associated with the use of ozone, such as sinus arrest, myocardial infarction, or vasospasm [10,11]. With enhanced exposure to ozone, erythrocytes are increasingly damaged. Thus, the application of appropriate doses of ozone in autohemotherapy for patients with different diseases would appear to be crucial.

It is well known that ozonation affects the functions of blood cells. Increased ATP (adenosine triphosphate) and 2,3DPG (2,3-diphosphoglycerate) concentrations in erythrocytes have been observed, as well as decreases in the pH of the cytoplasm. It has been hypothesized that lipid oxidation products stimulate bone marrow and increase the release of erythrocytes with enhanced concentrations of glucose-6-dehydrogenase [12].

Ozone therapy is also known to increase neutrophil phagocytic activity [13]. Moreover, H_2_O_2_ activates tyrosine kinase and consequently causes increased synthesis and secretion of proinflammatory cytokines: IFN-γ, TNF-a, and IL-8 [14,15,16]. However, ozonated autohemotherapy does not seem to affect NK-cell function, IFN-γ, TGF-β, IL-1α, IL-1β, IL-2, IL-4, IL-6, IL-10, IL-13, and CRP levels, platelet function, blood coagulation, von Willebrand factor, erythrocyte function, and surprisingly protein peroxidation [17]. Reactive oxygen species or lipid ozonation products activate a wide spectrum of genes and consequently upregulate synthesis of acute-phase proteins and cytokines responsible for immunomodulatory effects [14,18]. The application of ozone autohemotherapy induces the release of PDGF-B, TGF-β_1_, IL-8, and EGF from platelets [19]. Ozonated blood is able to activate endothelial cells through lipid peroxidation products and stimulates them to release NO [20]. NO is a known vasorelaxant which dilates vessel walls, having beneficial effects particularly in hypoxic lower-limb tissues or in the retina [21,22]. These effects seem to be mediated via activation of the NRF2 pathway [23,24,25]. 

Microparticles (MPs) are spherical fragments of cell membranes with diameters ranging from 0.1 to 1.0 μm, which are released from eukaryotic cells to the extracellular space. The formation of MPs occurs during maturation, activation, and apoptosis of a cell [26]. MPs consist of cell membrane lipids and proteins, as well as elements of cytoskeleton originating from the mother cell. The properties of MPs mainly depend on the factors initiating their release. Different stimulants induce the release of MPs, which present distinct properties even if they are released from the same type of cells. There is no cellular core in microparticles and they do not synthesize their own proteins; however, they may contain biologically active molecules from the cell of origin, i.e., enzymes, membrane proteins, mRNAs and microRNAs, adhesive proteins, coagulation factors, and membrane lipids [27,28,29,30]. All of these take part in the regulation and coordination of many processes occurring within the organism, i.e., inflammatory processes, neoangiogenesis, coagulation, and fibrinolysis [27,31,32,33,34,35]. By means of their role in transmitting signals between cells, microparticles constitute a connection between coagulation processes and local inflammation [28,29]. They also play a role in the progression of cancer [36]. MPs are able to modify the activity of certain medicines due to their sequestration [29]. To date, there are no data on the effects of ozone on MPs.

The aim of the study was to assess the possible impact of whole-blood ozonation on the release of erythrocyte-derived microparticles (ErMP), leukocyte-derived microparticles (LMP), endothelial-derived microparticles (EnMP), and platelet-derived microparticles (PMP), as well as on selected parameters of the hemostatic system.

## 2. Materials and Methods

### 2.1. Subjects 

Blood for experiments was obtained from 19 healthy men (25–35 years old) not taking any drugs, with no history of thrombosis, hypertension, or disorders of the endocrine system, and with similar basic blood morphology (RBC, WBC, PLC, and hemoglobin levels, as well as MHC). The selected group of donors was highly homogeneous in terms of age, gender, and hormonal factors. The presented study conforms with the principles outlined in the Declaration of Helsinki and was approved by the Ethical Committee for Human Studies of the Medical University of Bialystok (KB: R-J-002/78/2015); prior to the study, all participants gave their informed consent.

### 2.2. Blood Collection

Eighty milliliters of venous blood was drawn from an antecubital vein into a collection bag and mixed with citrate phosphate dextrose solution (0.12% final concentration). After collection, the blood was split into four equal parts and transferred into ozone-resistant (Teflon) blood bags (Fresenius Hemofreeze Bag DF 200, Fresenius, Germany). Each blood sample was assigned to one of four groups (C, A, O1, and O2) and was treated according to the conditions defining each of the groups. There were 19 portions of blood from each of the different donors in all of the groups. The control group (C) was not subjected to ozonation or aeration, the second group (A) was subjected to aeration, the third group (O1) was ozonated with a lower dose of ozone, and the fourth group (O2) was ozonated with a higher dose of ozone.

### 2.3. Blood Ozonation/Aeration

Blood samples from groups A, O1, and O2 were subjected to either aeration or ozonation. The oxygen–ozone gas mixture was freshly prepared from a medical-grade oxygen using an ATO-3 MINI ozone generator (CryoFlex, Poland). The mixture was kept at a normal atmospheric pressure.

The doses of ozone used in our study are highly comparable to doses used in ozonation protocols designed for therapeutic use [8,37]. Ozonation/aeration was performed immediately after blood collection and splitting. The blood samples were treated with a single dose of 15 µg/mL (group O1) or 30 µg/mL (group O2) of oxygen–ozone gas mixture (final concentrations of gaseous ozone) or the same volume of air (group A). The gas mixtures were transferred into the blood using syringes made of ozone-resistant material containing an antibacterial filter. Infusion pump-operated syringes were used in order to maintain a constant flow of 1 mL/min. Although an infusion pump may not be according to the ozonation protocol designed for therapeutic use, it helped to maintain standard conditions of experimental ozonation in our study. During ozonation/aeration, blood bags were placed on a laboratory shaker to ensure mixing of the gas with the blood. Three minutes after the treatment, blood samples were taken from the blood bag: 5 mL for MP analysis and 5 mL for coagulation tests. The samples for coagulation tests were centrifuged at 2000× *g* for 10 min to obtain plasma. All samples of plasma were stored at −80 °C until the day of analysis.

### 2.4. Microparticle Isolation 

Two steps in the process of centrifugation were used in order to isolate microparticles from whole blood. First, blood samples were centrifuged at 5000× *g* at 24 °C for 5 min without centrifuge brake (removal of blood cells) to prepare platelet-poor plasma (PPP). To collect MP, 600 µL of PPP was centrifuged at 17,000× *g* at 20 °C for 3 min, and the supernatant was removed. The MP pellet was then resuspended in 500 µL of diluted annexin V FITC binding buffer (BD bioscience, Franklin Lakes, NJ, USA).

### 2.5. Quantification and Characterization of MPs

Quantification of the number of MPs and their phenotypic characterization in the pellet were conducted by flow cytometry (Facscalibur, Becton Dickinson, USA). The MP size gate was set between 200 and 1000 nm using fluorescent latex beds 0.2, 0.5, and 1 µm (Precision Size Standards, Polysciences). The total number of MPs was defined as all the events falling within the MP gate. To count the number of MPs, an aliquot of resuspended MP pellet (100 µL) was added to TruCount (Becton Dickinson, USA) (100 µL), followed by counting up to 2000 0.5 µL bead components of TruCount (total absolute count of MPs = (events in region except beads/events in region of beads) × (absolute number of beads/µL/sample volume (µL)). The origin of MPs was determined by flow cytometry using the cell specific monoclonal antibodies (BD bioscience) and annexin V FITC as follows: erythrocyte-derived microparticles—GlyA PE CD235 (clone GAR-2 (HIR2)), platelet-derived microparticles—CD42bPE-Cy^TM^5 (clone HIP1), leukocyte-derived microparticles—CD45PerCP-Cy^TM^5,5 (clone TU116), endothelial-derived microparticles—CD144 PE (clone 55-7H1).

### 2.6. Coagulation Tests

APTT was measured using the Hemostat APTT-EL reagent. Measurement of the PT time was carried out with the use of Hemostat Tromboplastin SI reagent. Fibrinogen concentration was determined with the use of the Hemostat Fibrinogen kit based on the Clauss method. Concentration of D-dimers was determined using the qualitative test of the VIDAS system for immuno-enzymatic marking of fibrin degradation products with the use of the immuno-fluorescence technique (BioMerieux).

### 2.7. Statistical Analysis 

Data analysis was carried out using the GraphPad Prism 8.4.0 (Software LLC). Quantitative variables were described in terms of means, standard deviations, and correlation coefficients. Qualitative variables were described as a number and percentage. To compare parametric quantitative variables between two groups, the Friedman test was used. Multiple comparisons analysis was carried out with Dunn’s test. A *p*-value of <0.05 was considered significant in all analyses.

## 3. Results

### 3.1. Microparticles

**Figure 1:**
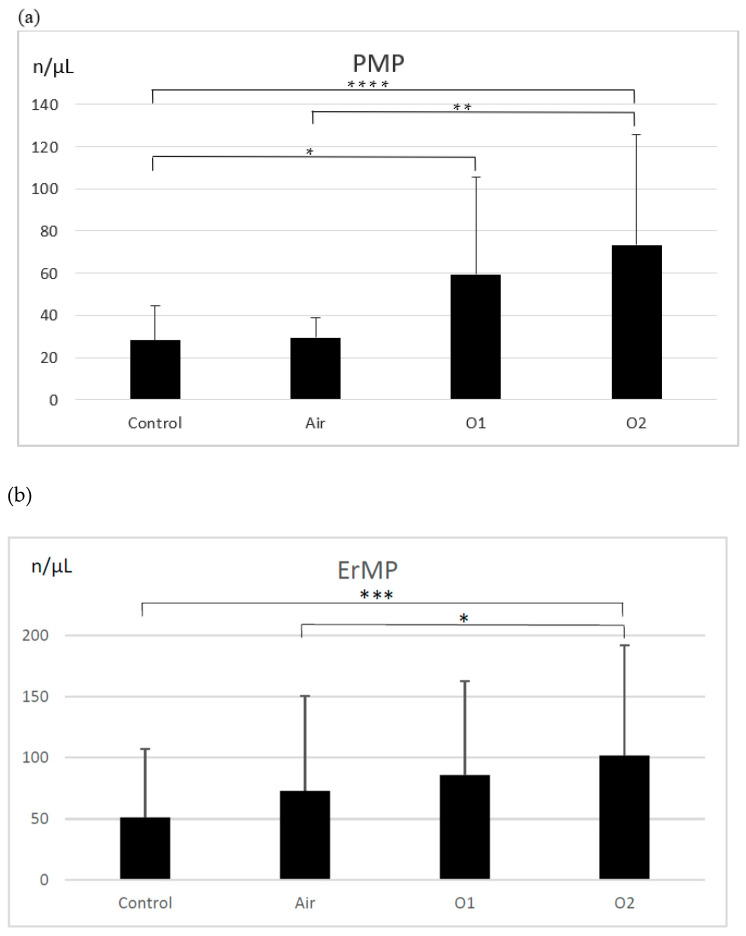

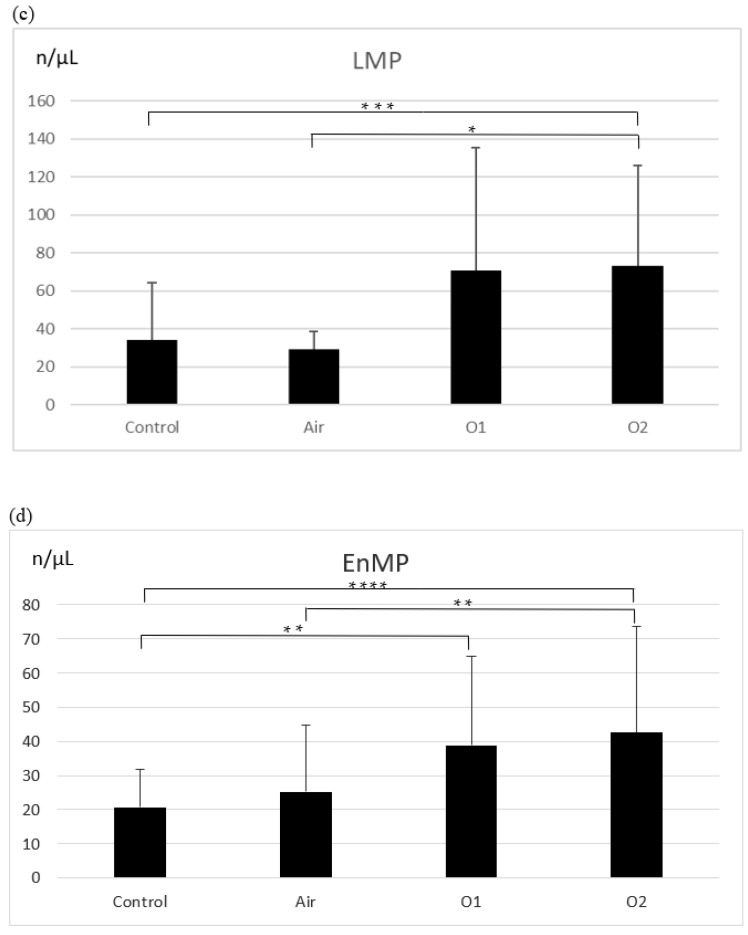
The effect of ozone on microparticle generation from platelets (**a**), erythrocytes (**b**), leukocytes (**c**), and endothelial cells (**d**). PMP—platelet-derived microparticles, ErMP—erythrocyte-derived microparticles, LMP—leukocyte-derived microparticles, EnMP—endothelial cell-derived microparticles, control—the control group (*n* = 19); Air—the aerated group (*n* = 19); O1—the group treated with ozone at a dose of 15 µg/mL (*n* = 19); O2—the group treated with ozone at a dose of 30 µg/mL (*n* = 19). Mean ± SD, statistically significant difference between the groups (* *p* < 0.01; ** *p* < 0.005; *** *p* < 0.0005; **** *p* < 0.0001).

There were more PMPs generated in the ozonated samples than in the control group (28.1 ± 16.2 MP/µL; *p* < 0.0001 for the group ozonated with higher dose and *p* < 0.01 for the group ozonated with a lower dose of ozone) (Figure 1a). The group treated with a higher dose of ozone generated significantly more PMPs than the aerated group (73.4 ± 52.5 MP/µL vs. 29.4 ± 9.5 MP/µL; *p* < 0.005). Aeration did not result in an increase in PMPs compared to controls. There were significantly more ErMPs generated in the group treated with a higher dose of ozone (102.0 ± 90 MP/µL) compared to the control group (51.3 ± 56.2 MP/µL; *p* < 0.0005) and the aerated group (73.0 ± 77.7 MP/µL; *p* < 0.01) (Figure 1b). The lower dose of ozone, as well as aeration, did not result in an increase in ErMPs compared to controls.

There were significantly more LMPs generated in the group ozonated with higher dose of ozone compared to the control group (34.3 ± 29.9 MP/µL; *p* < 0.0005) and the aerated group (24.4 ± 9.5 MP/µL; *p* < 0.01) (Figure 1c). The ozonation with the lower dose of ozone, as well as aeration, did not result in an increase in LMPs compared to controls. We observed more EnMPs generated in both ozonated groups compared to the control group. In the group treated with a higher dose of ozone, there were significantly more EnMPs than in the aerated group (42.6 ± 31.1 MP/µL vs. 25.2 ± 19.6 MP/µL; *p* < 0.005). The difference between both ozonated groups was not significant (Figure 1d).

Endothelial cells appear in very low numbers in the blood; hence, increased numbers of endothelial-derived MPs may seem surprising. We can only speculate on the mechanism responsible for this phenomenon. Possibly an increased number of EnMPs result from the fragmentation of the MPs already present and not the production of new MPs. Ultimately, it could be a question of an increase in specific surface area for the same specific volume. We cannot exclude the possible role of endothelial progenitor cells released into circulating blood from bone marrow. These cells also express CD144 on their surface and, thus, may generate the CD144^+^ microparticles detected in our study.

A representative light-scatter dot plot to show the MP region and representative fluorescence plots of labeled samples are presented in Figure S1 (control) and Figure S2 (ozonated blood) as well as the descriptive statistics of the obtained results is presented in Table S1 in Supplemental Material

### 3.2. Coagulation Tests

**Figure 2:**
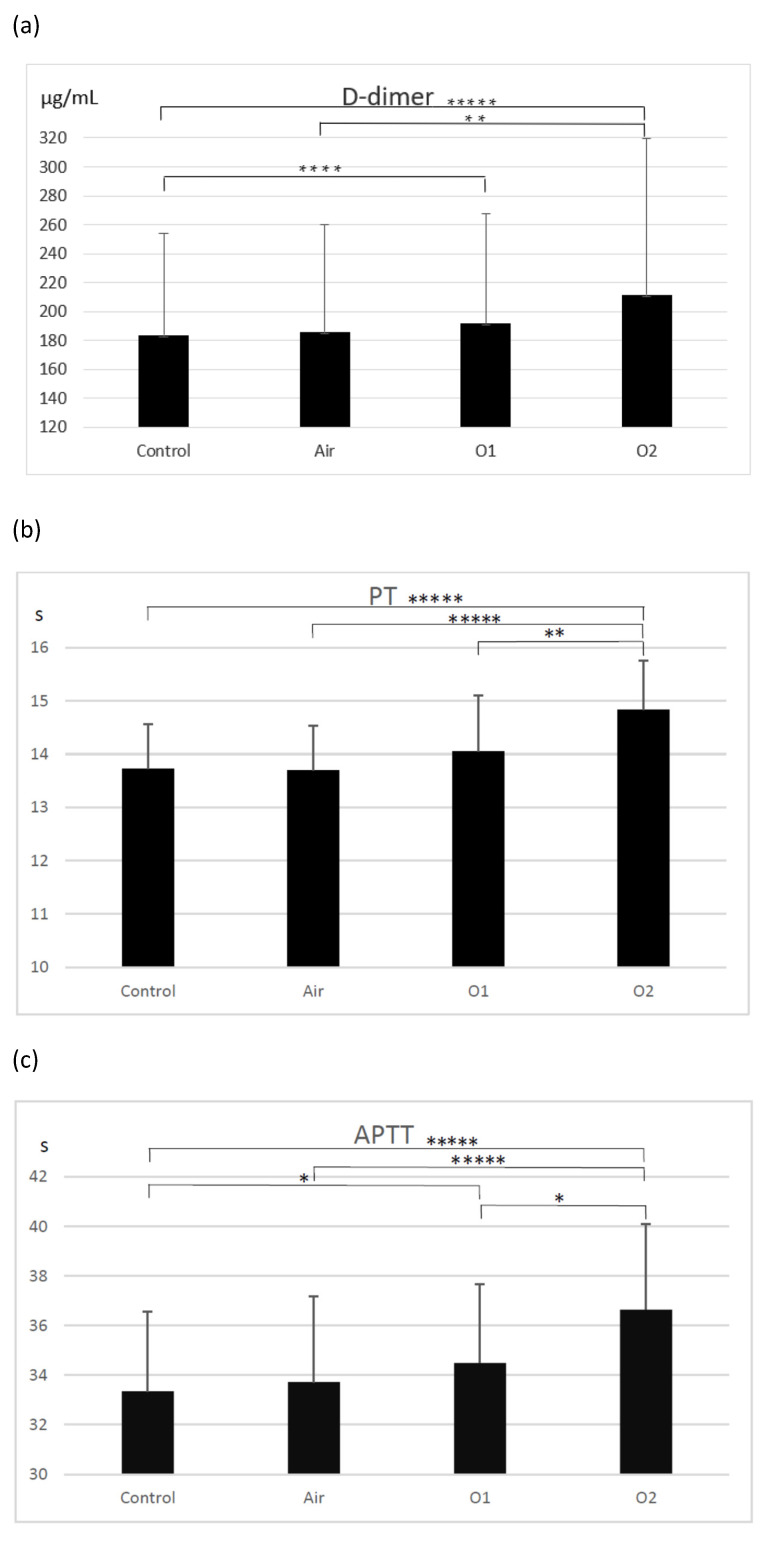

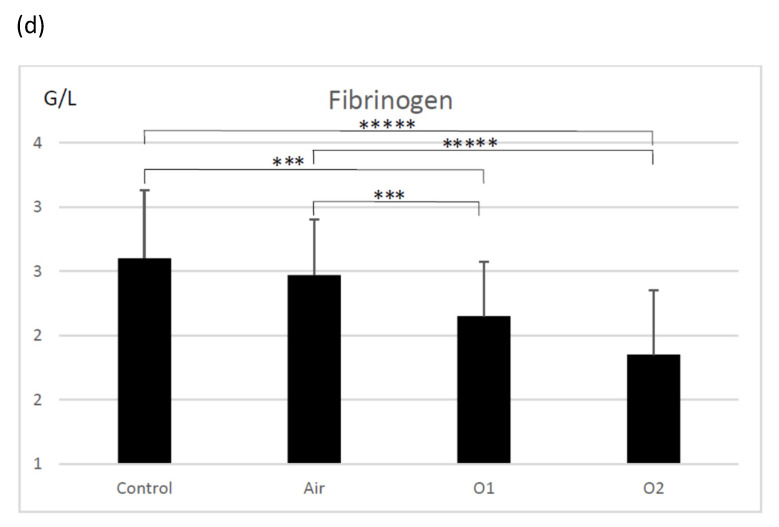
The effect of ozone on D-dimer concentration (**a**), PT—prothrombin time (**b**), APTT—activated thromboplastin time (**c**), and fibrinogen concentration (**d**). Control—the control group (*n* = 19); Air—the aerated group (*n* = 19); O1—the group treated with ozone at a dose of 15 µg/mL (*n* = 19); O2—the group treated with ozone at a dose of 30 µg/mL (*n* = 19). Mean ± SD, statistically significant difference between the groups (* *p* < 0.05; ** *p* < 0.01; *** *p* < 0.005; **** *p* < 0.0005; ***** *p* < 0.0001).

D-dimer molecules are produced by the breakdown of crosslinked fibrin during fibrinolysis. D-dimer generation requires the activity of thrombin, active factor XIII, and plasmin. The process begins when the thrombin formed by the activation of the coagulation system transforms soluble fibrinogen molecules into fibrin monomers. Elevated levels of D-dimer indicate the activation of coagulation and fibrinolysis. The concentrations of D-dimers in plasma in the investigated groups are presented in Figure 2a. An enhancement of D-dimers was observed in the ozonated samples (191.9 ± 75.8 µg/mL—lower dose, 211.6 ± 107.8 µg/mL—higher dose) compared to the control group (183.6 ± 70.2 µg/mL; *p* < 0.0001 for O2 group and *p* < 0005 for O1 group) and in the ozonated samples with the higher dose and the aerated group (186.1 ± 74.2 µg/mL; *p* < 0.01). The differences between the control group and the aerated group, as well as between both ozonated groups, were not significant.

The prothrombin time (PT) is a measure of the integrity of the extrinsic and final common pathways of the coagulation cascade. This consists of tissue factor, factors VII, II (prothrombin), V, X, and fibrinogen. Compared to the control group (13.7 ± 0.8 s), as well as to the aeration group (13.7 ± 0.8 s), there was a significant prolongation of PT observed in the samples ozonated with the higher dose of ozone (14.8 ± 0.9 s; *p* < 0.0001). There was also a significant prolongation of PT in the group ozonated with the higher dose of ozone compared to the group ozonated with the lower dose (*p* < 0.01). PT did not differ significantly across the control, aerated, and ozonated groups with the lower dose (14.1 ± 1.1 s) (Figure 2b).

The activated partial thromboplastin time (APTT) is a functional measure of the intrinsic and common pathways of the coagulation cascade and evaluates factors XII, XI, IX, VIII, and X, prothrombin, and fibrinogen. There was a prolongation of APTT observed in both groups treated with ozone (34.5 ± 3.2 s—lower dose, 36.7 ± 3.5 s—higher dose) compared to the control group (33.4 ± 3.2 s; *p* < 0.05) and the aerated group (33.7 ± 3.5 s; *p* < 0.05). APTT was also longer in the group treated with a higher dose of ozone compared to the group ozonated with a lower dose, as well as in comparison to the aerated group (*p* < 0.05) (Figure 2c).

Fibrinogen, a glycoprotein complex that circulates in the blood during tissue and vascular injury, is converted enzymatically by thrombin to fibrin and then to a fibrin-based blood clot. Compared to the control group (256.1 ± 53.8 mg/dL) and the aerated group (247.3 ± 43.1 mg/dL), there was a statistically significant decrease observed in fibrinogen concentration in both groups treated with ozone (215.1 ± 42.3 mg/dL—lower dose, 185.2 ± 49.6 mg/dL; *p* < 0.0001 for comparison with control group and *p* < 0.005 for comparison with the aerated group) (Figure 2d).

### 3.3. Correlations between Generated Microparticles of Different Origin 

In both groups treated with ozone, statistically significant correlations were noted between the generated microparticles: between ErMPs and LMPs (*r* = 0.7 and *r* = 0.79, respectively, for groups O1 and O2), between ErMPs and PMPs (*r* = 0.82 and *r* = 0.63, respectively, for groups O1 and O2), between ErMPs and EnMPs (*r* = 0.8 and *r* = 0.86, respectively, for groups O1 and O2), between LMPs and EnMPs (*r* = 0.7 and *r* = 0.76, respectively, for groups O1 and O2), between LMPs and PMPs (*r* = 0.75 only for group O1), and between PMPs and EnMPs (*r* = 0.78 and *r* = 0.76, respectively, for groups O1 and O2).

### 3.4. Correlations between the Generated Microparticles and Markers of Hemostasis

There were no statistically significant correlations demonstrated between the generated microparticles and APTT, PT, fibrinogen concentration, and D-dimers observed.

The descriptive statistics of the obtained results is presented in Table S1 in Supplemental Material.

## 4. Discussion

In a previous study, we evaluated the effects of ozone on human blood, using metabolomics [38]. After ozonation of the whole blood, a slight hemolysis (up to 0.6%) was observed. We found several metabolites, whose presence was clearly related to the ozone dose or which were present only in ozonated samples: bilirubin, biliverdin, dihydroxyvitamin D3, pyroglutamic acid, hydroperoxyoctadecadienoic acid (HPODE), trihydroxyoctadecenoic acid (Tri-HOME), and hydroxyoctadecatrienoic acid (HOTE). Few of these compounds are plasma antioxidants, with the exception of bilirubin and biliverdin, which have been shown to be potent antioxidants. Bilirubin acting as an antioxidant is oxidized to biliverdin, which may explain the increase in biliverdin and decrease in bilirubin concentrations observed after the treatment of blood with ozone. The concentration of pyroglutamic acid, which is a component of GSH metabolism and is involved in the plasma antioxidative system, was also decreased. The other metabolite differentiating ozonated samples from the others was dihydroxyvitamin D3. Vitamin D3 has already been shown to prevent lipid peroxidation.

There are no published data on the effect of ozone on microparticle release. Our study revealed increasing and dose-dependent MP release following ozonation, which may suggest that membrane fluidity is elevated within the studied dose range.

The present study provides the first evidence that ozonation of blood induces microparticle release from blood cells. Moreover, the number of released microparticles was proportional to the applied dose of ozone. Statistically, microparticles released from different blood cells and endothelial cells correlated significantly with each other, which allows us to assume that the effect of ozone is not cell-specific.

The observed release of microparticles raises an important question: what risk does it pose for patients transfused with ozonated blood? No pertinent data have been published so far. During preparation and storage of blood components, blood cells release microparticles which remain in the extracellular fluid and are finally transfused to the recipients [39]. The number and origin of the microparticles generated differ significantly according to the method and the time of preparation of blood components, depending also on the type of storage prior to separation into components and after that, before release for transfusion [40,41,42].

Due to the variation in methodologies used for isolation of microparticles and in the assessments of MPs by flow cytometry, it is very difficult to compare our data and the results of studies published so far, on the release of MPs from blood components [43]. We observed a twofold increase in the MPs derived from red blood cells and leukocytes after ozonation, while other investigators reported increased MP concentrations ranging from 6.8% [42] to 17% [44].

Microparticles in blood components are at least partially responsible for the immunomodulatory effect of transfusions [45]. This adverse reaction contributes to immunosuppression, cytokine inhibition, and stimulation of inflammatory reactions. Transfusion-related immunomodulation has been shown to have a favorable effect on exacerbations in the course of Crohn’s disease, as well as on successful kidney transplantation [46].

Microparticles in the blood components may also contribute to thromboembolic complications observed in the recipients of blood transfusions [45]. These microparticles take part in the processes of coagulation and fibrinolysis, bind coagulation factors [47], present tissue factor [27], and activate coagulation on their surface. MPs express receptors for activated protein C, an inhibitor inactivating coagulation factors Va and VIIIa [31].

In parallel, but not in statistical relation to the release of MPs, we demonstrated a statistically significant effect on the basic markers of hemostasis. We observed a prolongation of APTT and PT, a decrease in fibrinogen concentration, and an increase in D-dimer concentration. Activation of coagulation may be responsible for fibrinogen consumption; however, a direct effect of ozone on fibrinogen cannot be excluded. Rosenfeld et al. [48] revealed that ozone increases the number of hydroxyl and carbonyl groups on the fibrinogen molecule. D-fragments and, to a lesser extent, fibrinogen fragments are particularly sensitive to ozone-induced oxidation. Oxidation-induced fibrinogen modifications may affect its secondary structure and result in higher crosslinking potential. The present in vitro study used purified plasma; thus, we cannot extrapolate the results to autohemotherapy. Decreased fibrinogen concentration might be one of the possible mechanisms for the observed APTT and PT prolongation. Although statistically significant, the changes in APTT, PT, and fibrinogen were somewhat mild (APTT—9.8%, PT—8%, fibrinogen—27.7%) and did not exceed normal values. More detailed analyses of the effects of microparticles on blood coagulation have been published by other investigators [33,34,35,49,50]. While a coincidence of ozone-induced release of microparticles and changes in hemostasis markers was observed in our study, we did not find a statistically significant correlation among the parameters investigated.

Elevated D-dimer concentration reflects the activation of both coagulation and fibrinolysis. Some MPs are able to activate the release of plasmin, which suggests that the role of MPs in hemostasis is more complex than previously assumed [50]. Meijden et al. [33] showed that MPs released from erythrocytes and platelets are able to directly activate factor XII, which most probably explains the activation of fibrinolysis rather than coagulation. Direct activation of factors IX and XI by MPs points to the probable mechanism of activation of the tissue factor-dependent coagulation pathway [34]. Elevated concentration of MPs in ozonated blood and blood components may affect coagulation and fibrinolysis and increase thromboembolic risk [35]. It is important to emphasize that we studied the effect of ozone on blood in vitro, and that our results may explain some of the adverse reactions observed in patients treated with autohemotherapy using ozonated blood. Üreyen et al. [11] reported an occurrence of myocardial infarction after autohemotherapy in a patient with no risk factors for thromboembolic complications. Vasospasm of the left main coronary artery and total occlusion of the right coronary artery due to thrombus were observed in the patient. However, these findings should be treated with caution, as there is no direct proof that the autohemotherapy was responsible for the observed adverse effects [51]. MPs were not evaluated in the study. Nevertheless, during autohemotherapy with ozonated blood, the reinfusion of a quantity of ozone-treated blood (a maximum of 200 mL, compared with the approximately 5 L in circulation) in the same patient takes place drop by drop, thus allowing a buffer situation for any slight variations in the parameters linked to blood coagulation [52]. Moreover, some authors have published positive effects of systemic ozone administration in blood hemostasis parameters [52,53,54].

## 5. Conclusions

The present study provides evidence that blood ozonation induces the release of microparticles from erythrocytes, leukocytes, platelets, and endothelial cells in a dose-dependent manner. The observed weak, but statistically significant changes in the hemostasis system need further research, as preclinical and clinical papers with systemic ozone point in the opposite direction.

## Data Availability

Data is contained within the article and supplementary material.

## Supplementary Materials

The following supporting information can be downloaded at: https://www.mdpi.com/article/10.3390/biom12020164/s1, Figure S1: The representative light-scatter dot plot demostrating the MP region and representative fluorescence plots of labeled samples (control); Figure S2: The representative light-scatter dot plot demostrating the MP region and representative fluorescence plots of labeled samples (ozonated blood); Table S1: titleDescriptive statistics of the obtained results.
