# Supplementary material for: Ozonation of Whole Blood Results in an Increased Release of Microparticles from Blood Cells
Source: Biomolecules. 2022 Jan 21;12(2):164. doi: 10.3390/biom12020164 (PMC8961535; doi:10.3390/biom12020164)
Supplement: Supplementary file 1 [file biomolecules-12-00164-s001.zip › biomolecules-1473987-supplementary.pdf]

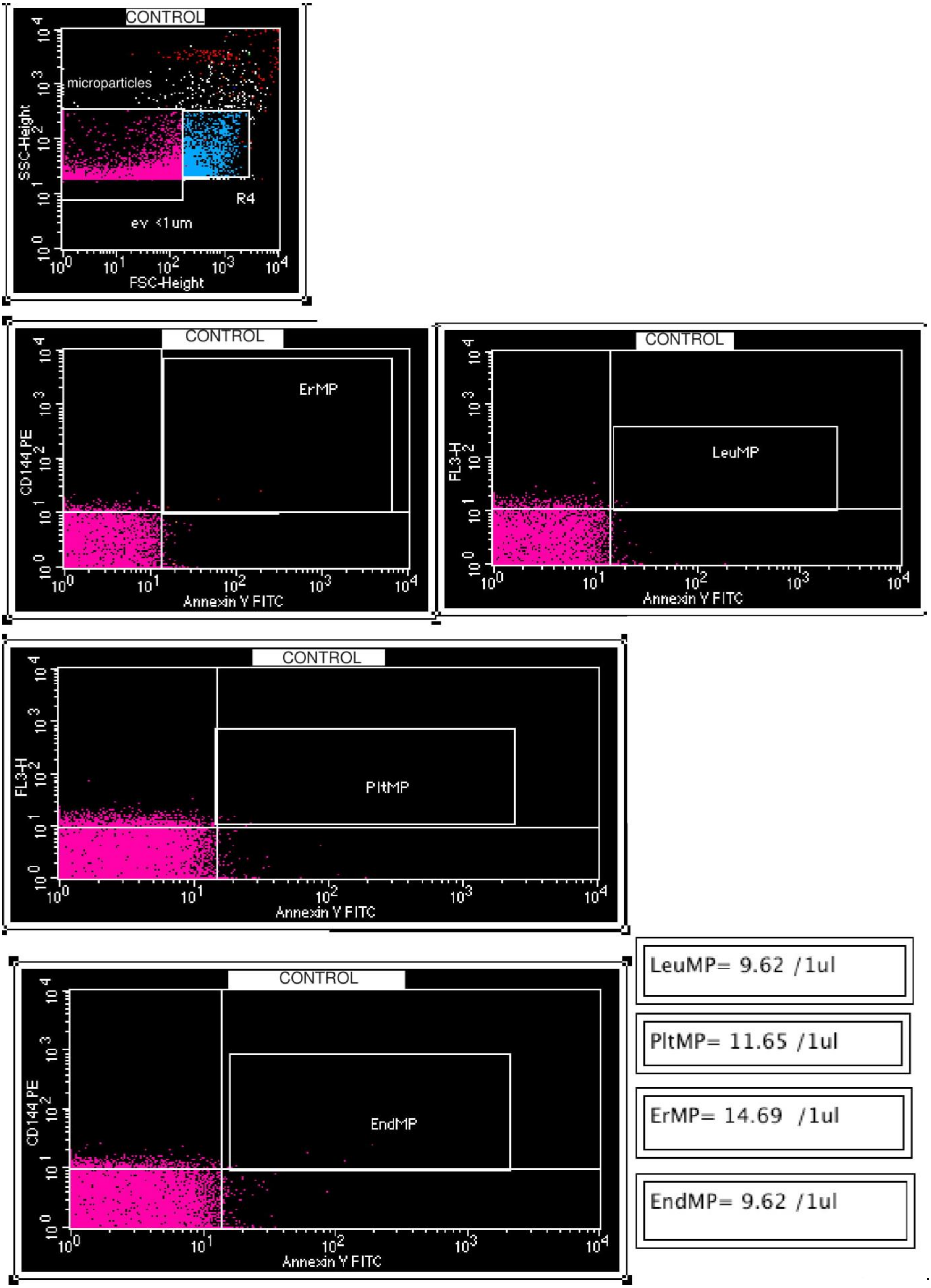

Figure S1. A representative light-scatter dot plot demonstrating the MPs region and representative fluorescence plots of labeled samples in the control sample.

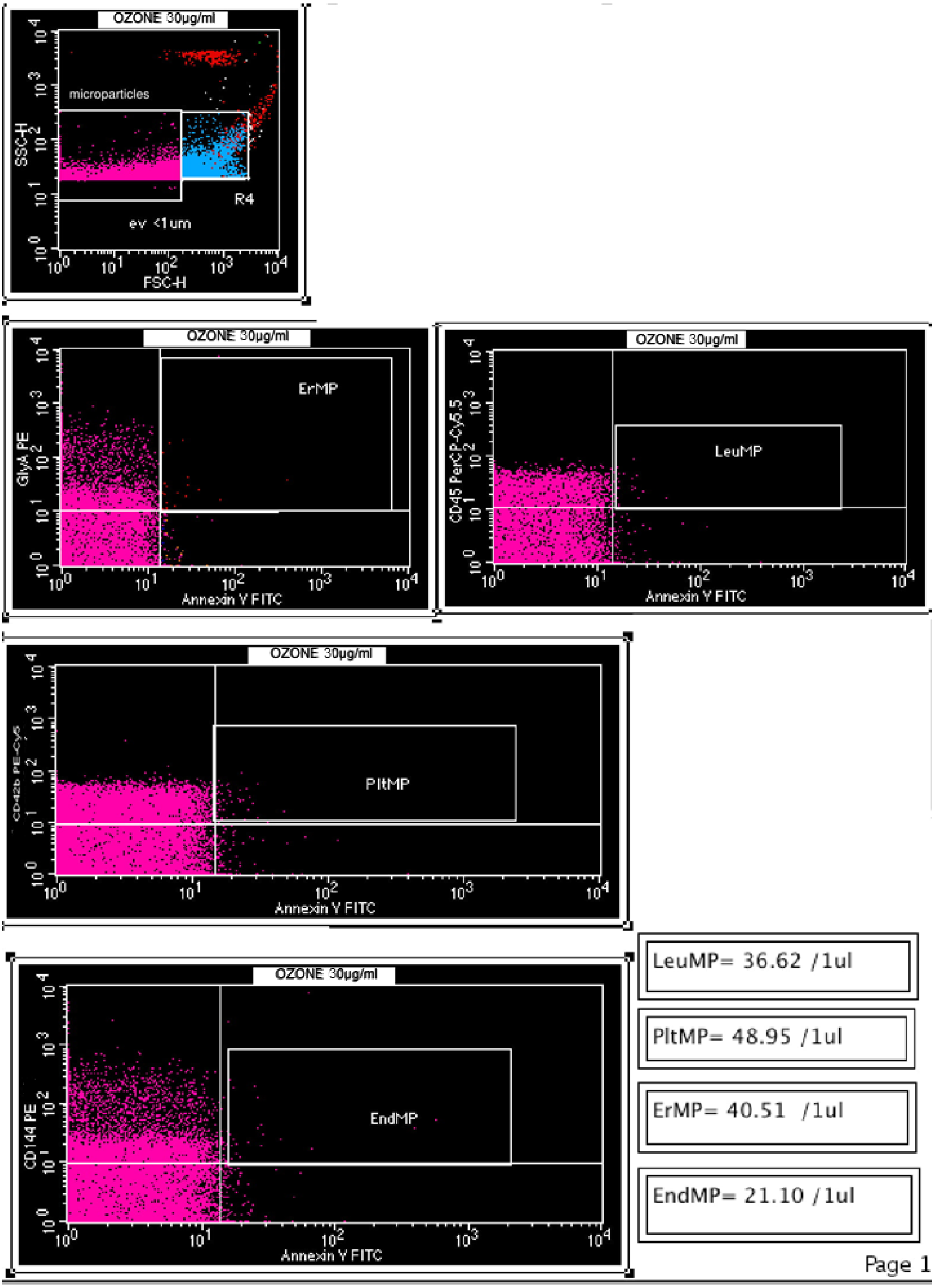

Figure S2. A representative light-scatter dot plot demonstrating the MPs region and representative fluorescence plots of labeled samples in a sample treated with a higher dose of ozone.

Table S1. Descriptive statistics of the obtained results. ErMP – erythrocyte-derived microparticles; LMP – leukocyte-derived microparticles; PMP – platelet-derived microparticles; EnMP – endothelial cells-derived microparticles; C – control group; A – aerated group; O1 – ozonated group with a dose of 15 μg/mL of ozone; O2 – ozonated group with w dose of 30 μg/mL of ozone.

		Mean	Median	Minimum	Maximum	SD
**D-dimers (μg/mL)**	C	183.60	175.00	74.00	334.00	70.20
	A	186.05	171.00	74.00	347.00	74.19
	O1	191.85	182.00	77.00	375.00	75.83
	O2	211.55	201.00	72.00	558.00	107.79
**PT (seconds)**	C	13.73	13.60	12.30	15.40	0.83
	A	13.70	13.65	12.40	15.70	0.84
	O1	14.04	14.15	11.60	15.80	1.06
	O2	14.83	14.65	13.60	16.50	0.92
**APTT (seconds)**	C	33.35	32.90	26.30	40.60	3.21
	A	33.72	33.20	26.70	41.60	3.45
	O1	34.50	34.50	28.10	41.50	3.16
	O2	36.64	36.25	30.40	43.10	3.45
**Fibrinogen (G/L)**	C	256.05	247.00	177.00	417.00	53.77
	A	247.30	242.50	179.00	353.00	43.06
	O1	215.05	204.50	162.00	341.00	42.34
	O2	185.15	175.00	116.00	305.00	49.59
**ErMPs (n/μL)**	C	51.25	37.00	12.00	200.00	56.21
	A	73.00	38.50	15.00	275.00	77.66
	O1	85.94	51.00	25.00	294.00	76.64
	O2	102.00	62.50	24.00	384.00	90.00
**LMPs (n/μL)**	C	34.29	28.00	4.00	91.00	29.89
	A	29.44	32.00	19.00	147.00	9.49
	O1	70.88	40.00	23.00	201.00	64.54
	O2	73.44	56.50	22.00	310.00	52.53
**PMPs (n/μL)**	C	28.13	26.00	10.00	133.00	16.21
	A	29.44	29.50	15.00	106.00	9.49
	O1	59.56	41.00	19.00	252.00	46.04
	O2	73.44	67.00	20.00	247.00	52.53
**EnMPs (n/μL)**	C	20.71	17.00	8.00	46.00	11.13
	A	25.19	19.50	9.00	129.00	19.61
	O1	38.88	31.00	10.00	118.00	26.02
	O2	42.63	33.00	15.00	201.00	31.01
